# Induction of Osteogenic Differentiation of Adipose Derived Stem Cells by Microstructured Nitinol Actuator-Mediated Mechanical Stress

**DOI:** 10.1371/journal.pone.0051264

**Published:** 2012-12-07

**Authors:** Sarah Strauß, Sonja Dudziak, Ronny Hagemann, Stephan Barcikowski, Malte Fliess, Meir Israelowitz, Dietmar Kracht, Jörn W. Kuhbier, Christine Radtke, Kerstin Reimers, Peter M. Vogt

**Affiliations:** 1 Department of Plastic-, Hand- and Reconstructive Surgery, Hannover Medical School, Hannover, Germany; 2 Laser Zentrum Hannover e.V., Hannover, Germany; 3 Technical Chemistry I and Center for Nanointegration University of Duisburg-Essen, Essen, Germany; 4 Biomimetics Technologies Inc., Toronto, Canada; Instituto Butantan, Brazil

## Abstract

The development of large tissue engineered bone remains a challenge in vitro, therefore the use of hybrid-implants might offer a bridge between tissue engineering and dense metal or ceramic implants. Especially the combination of the pseudoelastic implant material Nitinol (NiTi) with adipose derived stem cells (ASCs) opens new opportunities, as ASCs are able to differentiate osteogenically and therefore enhance osseointegration of implants. Due to limited knowledge about the effects of NiTi-structures manufactured by selective laser melting (SLM) on ASCs the study started with an evaluation of cytocompatibility followed by the investigation of the use of SLM-generated 3-dimensional NiTi-structures preseeded with ASCs as osteoimplant model. In this study we could demonstrate for the first time that osteogenic differentiation of ASCs can be induced by implant-mediated mechanical stimulation without support of osteogenic cell culture media. By use of an innovative implant design and synthesis via SLM-technique we achieved high rates of vital cells, proper osteogenic differentiation and mechanically loadable NiTi-scaffolds could be achieved.

## Introduction

Nitinol (NiTi) is a promising material in the field of innovative bone implants. Its mechanical characteristics are closer to those of bone than titanium, stainless steel, ceramics or any other material on the market [1], [2], [3]. The stiffness mismatches between implant materials and bone tissue in known to cause implant loosening [4]. The stiffness mismatch between NiTi and bone tissue is smaller than other and reduces tension.

However, the mechanical benefits of NiTi alone do not solve general problems of dense implants like:

long time for integration of implant in the surrounding bone tissue
insufficient/inadequate implant integration
need for bone cement or other filling materials
inflammatory reactions

Different implant design and processing offers an easier way to create customized osteoimplants: An important part of an innovative design is a so called bioactivation of the implant by presettlement with autologous adipose derived stem cells (ASCs). The basic idea by that is to shorten time to osseointegration und to reduce foreign body-reactions. Bruder et al. showed that presetteling of ceramic implants with mesenchymal stem cells (MSCs) leads to faster osseointegration [5]. Habijan examined the behavior of MSCs from bone marrow aspirates on NiTi and found it to be compatible with these cells [6], [7]. Compared to MSCs ASCs are easy available with minimal donor morbidity. The cells have the potential to undergo osteogenic differentiation [8], [9] and may lead to faster osseointegration. Best donor sites for ASCs are the inner tight and the lower abdomen as the highest concentrations of these cells are found there [10]. Harvesting can be done by syringe aspiration without general anesthesia which was shown to be a relatively less traumatic method [10].

As a dense metal-implant offers a comparatively small and plane surface for cell adhesion implant loosening may occur earlier than in sponge form implants where cells can grow in and which offers a bigger implant surface. Several studies already showed the suitability for the use of Nitinol as osteoimplant material with focus on porous shapes [11], [12], [13]. But when pores are too small cells stop ingrowth at the point of to less oxygen and/or food supply. For thicker cell layers and deeper ingrowth structures with interconnected pores are needed in vitro [14]. Porous NiTi is usually processed by techniques like injection molding. This requires the use of space holders to create pores. The generated pores and their interconnections are randomized. As a result there is no control for processing of optimal structures for cell ingrowth. Selective Laser Melting (SLM) of NiTi-microparticles is an alternative fabrication method which allows controlled processing.

The implant evaluated in the presented study is designed as a 3-dimensional (3-d) mesh structure which offers sufficient space for cell ingrowth, oxygen and food supply by diffusion in the early phase of implant integration and in later phase space for angiogenesis.

Meshes are produced by SLM of NiTi powder [15], [16]. This kind of processing allows synthesizing almost any 3-d structures designed by CAD (computer aided design) with a web thickness down to 50 µm. Knowledge about biocompatibility of laser modified NiTi is still limited. NiTi nanoparticles generated by ultrashort laser pulses were biocompatible to ASCs [17] and only toxic to endothelial and smooth muscle cells at very high concentrations [18]. Also treatment with longer pulses (cutting with a microsecond laser) does not affect the composition of NiTi [19]. These studies used short laser pulses in order to avoid heat deposition. In contrast, SLM uses continuous wave laser with intended heat flux in order to melt the particles surfaces.

Up to now, no data are available concerning the interaction of ASCs with NiTi fabricated by SLM. The study at hand checked out the toxic potential of SLM NiTi-structures as well as cell-surface interactions and cell morphology on the material in general. Furthermore the osteogenic differentiation capacities of ASCs on SLM NiTi structures were analyzed. Induction of differentiation by cell culture medium was compared to application of mechanical stress by implant compression.

## Methods

### Processing of NiTi-structures

Powder was generated by gas atomization from NiTi bar material. The size of powder particles ranges between 25 and 45 µm.

NiTi-meshes and complex 3-dimensional structures were assembled by selective laser melting as previously described [16], [20] with web width between 100 and 150 µm and a mesh size between 150 and 400 µm.

For cell culture NiTi-structures were disinfected with 70% Ethanol for 2 days. Loose powder was removed by ultrasonic bath in 70% ethanol 3×15 min. Afterwards structures were stored in sterile distilled water at 4°C.

### ASC-isolation and Cultivation

Human ASCs were isolated from fat tissue obtained from male and female donors aged between 16 and 40 years with the written informed consent from the patients as previously described [21] following ethical standards. According to the decision of the local ethics committee of Hannover Medical School, where the study was performed, no statement was needed for the use of human primary cells in this study. The received tissue was used anonymously. Fat was separated from the dermo-epidermal layer, minced with sterile scissors followed by enzymatic digestion with 0.075% collagenase CLS (Biochrom AG, Berlin, Germany) in PBS (Biochrom) for 30 minutes at 37°C under permanent shaking. Cell containing liquid was centrifuged at 1.200×g at room temperature for 10 minutes. Cells were cultivated in DMEM/Hams-F12 (PAA) with 10% fetal calf serum (FCS) (Biochrome), 50 U/ml penicillin/streptomycin (Biochrome), 10 ng/ml FGF and 50 µg/ml ascorbate-2-phosphate (Sigma Aldrich, Taufkirchen, Germany) at 37°C and 5% CO_2_. For analyses cells in passage 3 to 5 were seeded on cover glasses and Nitinol-surfaces with and without nanoparticle-coatings. Cultivation time varied according to analyses between 24 hrs, 48 hrs and 6 weeks (for osteogenic differentiation).

### Chemically Induced Osteogenic Differentiation

10^5^ cells each were seeded on variable NiTi-meshes or cage-like structures and cultivated in 24 well-plates (CytoOne) for 5 days. Osteogenic differentiation was induced by application of dexamethasone and glycerol-2-phosphate disodium salt. Cells were cultivated with DMEM/Hams-F12 with 10% FCS, 50 U/ml penicillin/streptomycin, 50 µg/ml ascorbate-2-phosphate, 39.3 ng/ml dexamethasone and 756 µg/ml glycerol-2-phosphate disodium salt up to 6 weeks. Medium was changed two to three times per week.

### Mechanically Induced Osteogenic Differentiation

**Figure 1:**
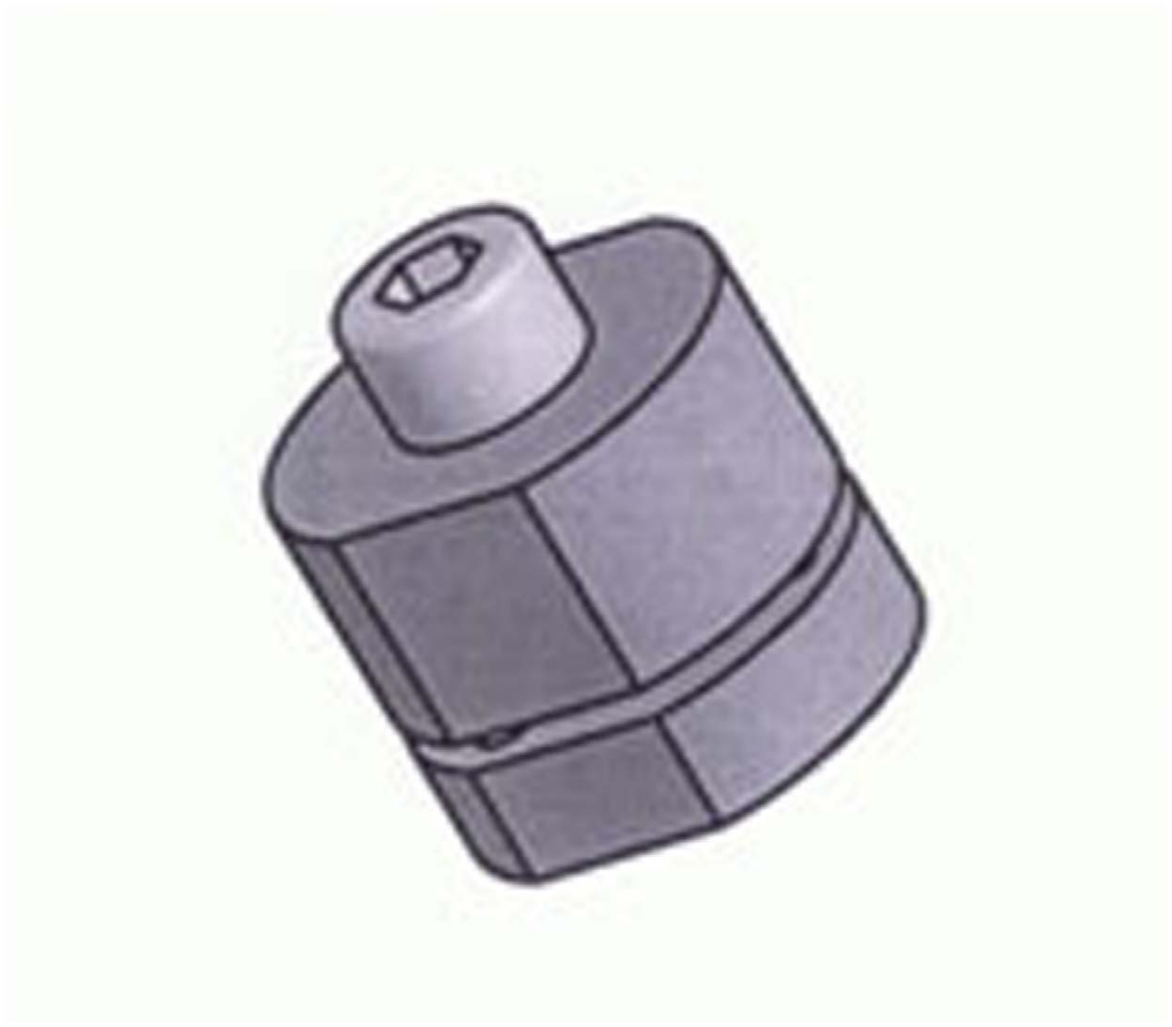
Titanium clamp for mechanical induction of osteogenic differentiation.

10^5^ Cells were seeded on 3-dimensional NiTi-structures and cultivated in 24 well-plates for 10 days. Osteogenic differentiation was induced by application of mechanical stress. Therefore three NiTi-cages were placed in Titanium clamps (Fig. 1) and exposed to compression. Compression was controlled by clamp screws, which were tightened with a torque wench between 5 and 20 Nm. Structures with cells were cultivated with compression in 6 well-plates containing 4 ml ASC-culture medium for 6 weeks.

### RAW264.7 Cultivation

The murine macrophage cell line RAW264.7 (ATCC, Manassas, USA) was cultivated with DMEM high glucose containing 10% FCS, 50 U/ml penicillin/streptomycin and 0.1 mg/ml sodium-pyruvate (PAA) at 37°C and 5% CO_2_.

### Cell Viability

Cell viability was tested by live/dead assay (Invitrogen, La Jolla, USA, #L3224) and cell titer blue assay (Promega, Mannheim, Germany, #8082).

For live dead assay ASCs were seeded on NiTi-structures and cultivated for 24–48 hrs (short time analyses of undifferentiated cells) or up to 6 weeks (long term analyses of osteogenic differentiated cells). To perform the assay cells were incubated with live/dead solution at 37°C for 30 min and then immersed in PBS analyzed by fluorescence microscopy (Olympus CK40, Cell F software). Living cells were stained green by calcein, dead cells red by ethidium-homodimer-1.

Cell titer blue assay for analyses of cells metabolic activity was performed in 96 well-plates. 10000 cells were cultivated with NiTi sheets (2×2 mm) (Alloy M, Memory Metalle GmbH, Weil am Rhein, Germany) for 24 to 48 hrs before cell titer blue solution was added and incubated at 37°C for 4 hrs. Cells metabolize resazurine via reduction to resorufin with an emission of 590 nm. The produced fluorescence is proportional to the number of viable cells. Fluorescence was read out with Tecan GENios multi well reader (Tecan, Männedorf, Swiss). Statistical analysis (one way ANOVA, followed by Dunnetts post hoc test) was performed with Graph Pad Prism Software.

### Microsections

As NiTi specimens are too hard for paraffine or cryo cross-sections, diamond cuts in acrylate had to be performed. For microsections 10000 cells were seeded on NiTi-structures and cultivated from 24 hrs up to 6 weeks with ASC-culture medium or osteogenic medium. Specimens were fixed in 3.7% formaline (Sigma Aldrich, Taufkirchen, Germany) and embedded in Technovit 7200 (Kulzer, Werheim, Germany) as specified by the manufacturer: dehydratation with ascending set of ethanol (30, 50, 70, 90, 95, 100%) for 3 hrs each, infiltration with ascending set of technovit 7200/ethanol (30, 50, 70, 100%) for one day each. Infiltrated specimens were embedded in technovit 7200, polymerization was started by white light in a vacuum (Histolux-Lichtbad, Hereaus, Wehrheim, Germany). Microsections of 30–50 µm thickness were stained with 0.5% methyleneblue for 30 min or haemalaun for 4 hrs and eosin for 50 min, then rinsed with distilled water, followed by washing with 80% and 96% ethanol. As last step samples were air dried and mounted with Technovit 7200.

### Immunofluorescence

Immunofluorescence was performed with ASCs seeded on NiTi-surfaces and incubated for 48 hrs up to 6 weeks and then fixed with 4% paraformaldehyde for 20 min, blocked and permeabilzed for 3×5 min with PBS containing 0.1% TritonX-100 and 1% BSA. Primary antibodies (table 1) were incubated for 1 h at room temperature in a wet chamber. The samples were washed with PBS containing 0.1% TritionX-100 and 0.1% BSA. Secondary antibodies (table 1) were incubated for 30 min at room temperature in a wet chamber. Samples were washed several times with PBS. Nucleic staining was done with DAPI supplemented mounting medium (Vectorshield #h-1200). Samples were analyzed by fluorescence microscopy (Zeiss Axiovert 200 M; Olympus SZX16, CellF).

**Table 1:** List of antibodies.

Antibody	Dilution	Manufacturer	product ID
bone morphogenic protein-2	1∶200	Abcam	ab14933
bone morphogenic protein-6	1∶200	R&D Systems	BAF507
bone alkaline phosphatase	1∶500	abcam	ab108337, ab17272
osteocalcin	1∶500	Abcam	ab13420
osteopontin	1∶1000	Abcam	ab8448
sparc	1∶1000	Abcam	ab14174
fibronectine	1∶200	Sigma	
collagen type I	1∶500	Abcam	ab292
collagen type I	1∶100	Chemicon	AB755P
Alexa Fluor 488	1∶1000	Invitrogen	A21200
Alexa Fluor 488	1∶1000	Invitrogen	A11008
Alexa Fluor 546	1∶4000	Invitrogen	A10040
Alexa Fluor 546	1∶4000	Invitrogen	A10036

### Alizarin Red Staining

Calcium inclusions in cells, which are characteristics of bony tissue, were detected by alizarin red staining. ASCs were seeded on NiTi-structures and differentiated osteogenic (chemically or mechanically) for 6 weeks. Samples were fixed with 3.7% formalin. Staining was performed following this procedure: rinse with tab water for 1 h, wash with 0.1 M boric acid (pH 4), incubated for 1 h in alizarin red solution (0.5%, pH 4), rinse with boric acid, rinse with distiiled water, wash with 95% ethanol, air dry. Calcium inclusions are stained red. Samples were analyzed by reflected-light microscopy (Olympus SZX16, Cell F) and digital microscopy (VK-9700 and VHX, both Keyence with VK- and VHX-Analyser Software).

### Scanning Electron Microscopy

Cells were seeded on NiTi-structures and cultured for 24 hrs and up to 6 weeks for osteogenic differentiation. Samples were then fixed in 0.2 M Na-cacodylate buffer (pH 7.4) with 2.5% glutaraldehyde for 24 hrs. Samples were incubated in 30, 50, 70, 90 and 100% acetone for 3×10 min. Critical point drying was performed with acetone/CO_2_ in CPD 030 system (Bal-Tec, Balzers, Liechtenstein). Samples were sputtered with gold (Sem Coating System, Polarion). Scanning electron microscopy was done with a SEM 505 (Philips). Pictures were taken with SEM-Software by Preiss and Gebert [22].

## Results

### Structure Design

**Figure 2:**
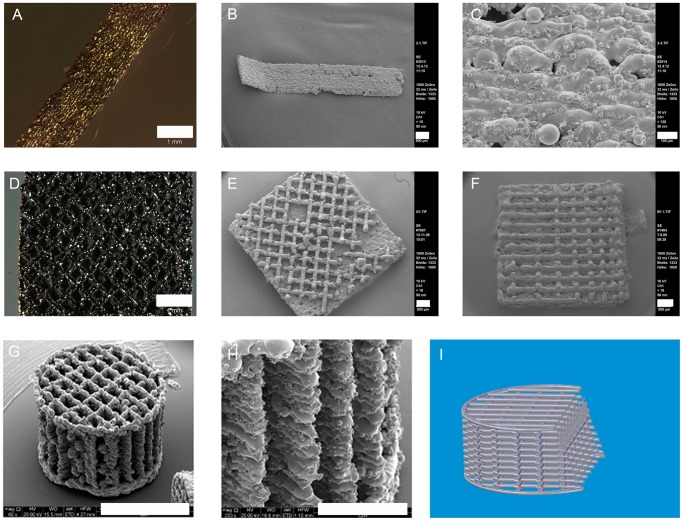
SLM-NiTi-structures: flat shaped (A–C), mesh-like (D–F), cage-like with inner structure (G–I); scale bars: 1 mm (A, D), 500 µm (B, C, E, F, H) and 2 mm (G); 2I draft of inner structure, created with Solid Edge (Siemens PLM Software).

We started our analysis by designing differently structured SLM NiTi devices of NiTi powder. Rationale behind the design was to create an optimized scaffold for ASC attachment and long-term growth. Basic investigation were performed with an unstructured NiTi slide of 2.5 mm width and 10 mm length without any pores or other surface structures (Fig. 2A and B) beside the roughness created by the laser melting process (Fig. 2C). ASCs have been described to prefer attachment to porous materials so meshes were processed in two different designs. In the first we created regular spaces (Fig. 2D and E) whereas in the second a screening of optimized gap width was performed (Fig. 2F). In three-dimensional cages the determined gap width with the best growth behavior of cells (Fig. 2F) were combined with an inner stacked mesh composition (Fig. 2G) and outer strength support (Fig. 2H). Here, a sufficient interconnection of pores (Fig. 2I) combined with mechanical stability could be achieved and such enough space for cell ingrowth, metabolic exchange and angiogenesis [14].

### SLM-generated NiTi is not Toxic to Cells

General cytocompatibility of NiTi was confirmed by viability tests with cell titer blue assay, using macrophages and ASCs. In case NiTi exerts an influence on the cells measured activity will be lowered as cells are no longer able to metabolize the contents of testing solution. Higher metabolic activity of macrophages can be a hint for macrophage activation and that there is an immune reaction to NiTi.

**Figure 3:**
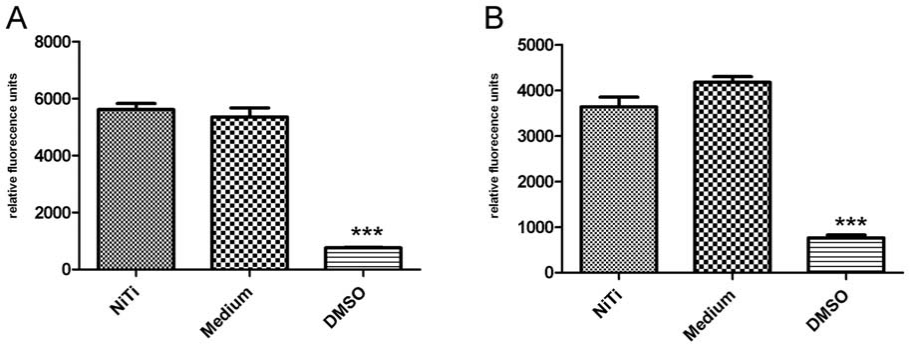
Metabolic activity of ASCs (A) and murine macrophages (B) on NiTi after 48 hrs of cultivation.

24 or 48 hrs ASCs grown under standard cell culture condition showed no significant differences in metabolic activity compared to cells grown on NiTi sheets excluding any harmful influence of the material itself. 10% DMSO, which is toxic to cells, served as toxic control and showed a significant down regulation of metabolic activity as cells died (Fig. 3A). Murine macrophage cell line raw264.7 also showed no significant reaction on stimulation for 24 or 48 hrs with NiTi in cell titer blue assay. Only the 10% DMSO control led to a significant down regulation of activity. Activation was not observed (Fig. 3B).

### Influence of SLM NiTi on ASCs

**Figure 4:**
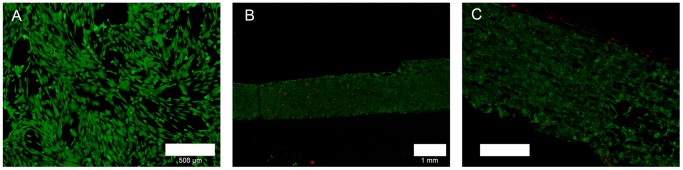
ASCs vitality on glass (A) and SLM-NiTi (B and C); green: vital cells, red: dead cells; scale bars 500 µm (A, C), 1 mm (B).

As this is the first study investigating compatibility of ASCs with SLM NiTi surfaces we started with a characterization of their vitality on SLM-NiTi. The devices were seeded with ASCs as described materials and methods and kept on them for 48 hrs in short-term culture and six weeks in long-term culture respectively. Unstructured glass surfaces were used as a positive control (Fig. 4A). Cells adhered to the NiTi slides comparable to the glass controls (Fig. 4B and C) and had a healthy spindle-shape appearance typical for ASCs. Rates of vital cells were nearly comparable to control cells settled on cover glasses with slightly more red fluorescent nuclei visible which indicates cell death (Fig. 4C). Same observations were made after long-term culture (data not shown) indicating the cytocompatibility of the SLM NiTi.

**Figure 5:**
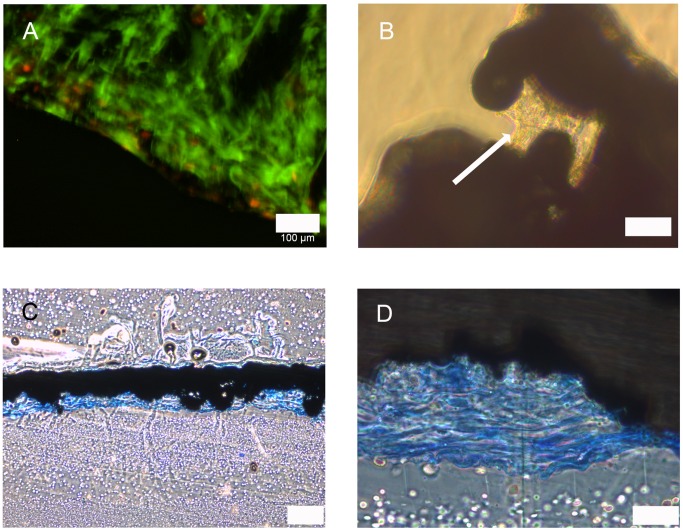
ASCs attachement to gap and web structures. A: live/dead staining; green: vital cells, red: dead cells; scale bar 100 µm. B: light microscopy; ASCs spanning between gap (indicated by arrow); scale bar 50 µm. C: acrylate microsection, methyleneblue staining; blue: ASCs; scale bar 200 µm. D: detail of C; scale bar 50 µm.

**Figure 6:**
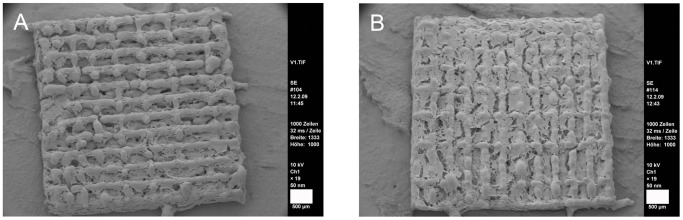
SEM of ASCs on meshes. A: after 48 hrs of cultivation; scale bar 500 µm. B: after one week of cultivation; scale bar 500 µm.

Cell attachment to the gap and web structured meshes was efficient. Cells ensheated the webs completely and grew inside the structures (Fig. 5A). Red fluorescence was observed but from this kind of analysis it was not clear whether this was a noise signal by the scaffolds or depending on cell death. Light microscopy pictures and microsections confirmed these findings (Fig. 5B–D). Cells started to extent between the webs (Fig. 5B) and grew in several ordered layers (Fig. 5C and D). In SEM analysis cells showed the expected ASC morphology and grew efficiently on the ligaments of the meshes (Fig. 6A). In a one week follow-up the cells overgrew the meshes’ gaps and formed thick layers. Extracellular matrix was secreted as clearly visible by the rough surface appearance (Fig. 6B).

### ASCs Differentiate Osteogenic on SLM-NiTi

**Figure 7:**
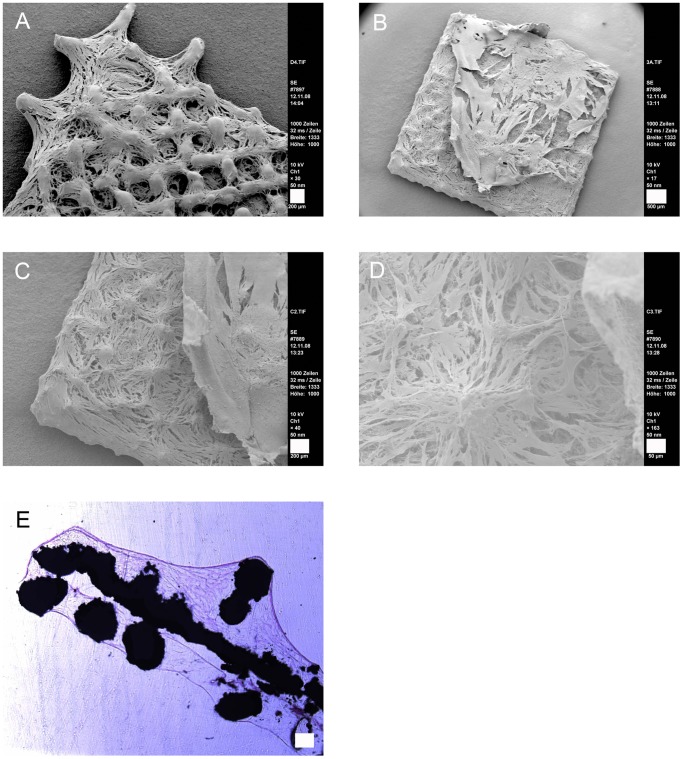
Osteogenic ASCs on SLM-NiTi meshes. A: after 3 weeks of osteogenic differentiation; scale bar 200 µm. B–D: after six weeks of osteogenic differentiation, scale bars 500 µm (B), 200 µm (C), 50 µm (D). E: microsection after six weeks of osteogenic differentiation; H&E staining; scale bar 200 µm.

ASCs’ osteogenic differentiation on NiTi was be induced chemically or mechanically as described in material and methods. We were interested in the osteogenic differentiation capacity of ASCs grown on SLM-NiTi taking into account the influence of the spatial cell arrangements on meshes and even more importantly on the three-dimensional cages. First a well established protocol of chemical induction was used for cells grown on meshes. Meshes were then analyzed by SEM after three (Fig. 7A) and six weeks (Fig. 7B–D). After chemical induction of osteogenic differentiation, ASCs secrete a thick extracellular matrix (ECM). Especially after six weeks the original mesh structure was no longer visible (Fig. 7B–D). For differentiation between superficial deposition of ECM and complete ingrowth we additionally performed H&E stainings on acrylate microsections (Fig. 7E).

**Figure 8:**
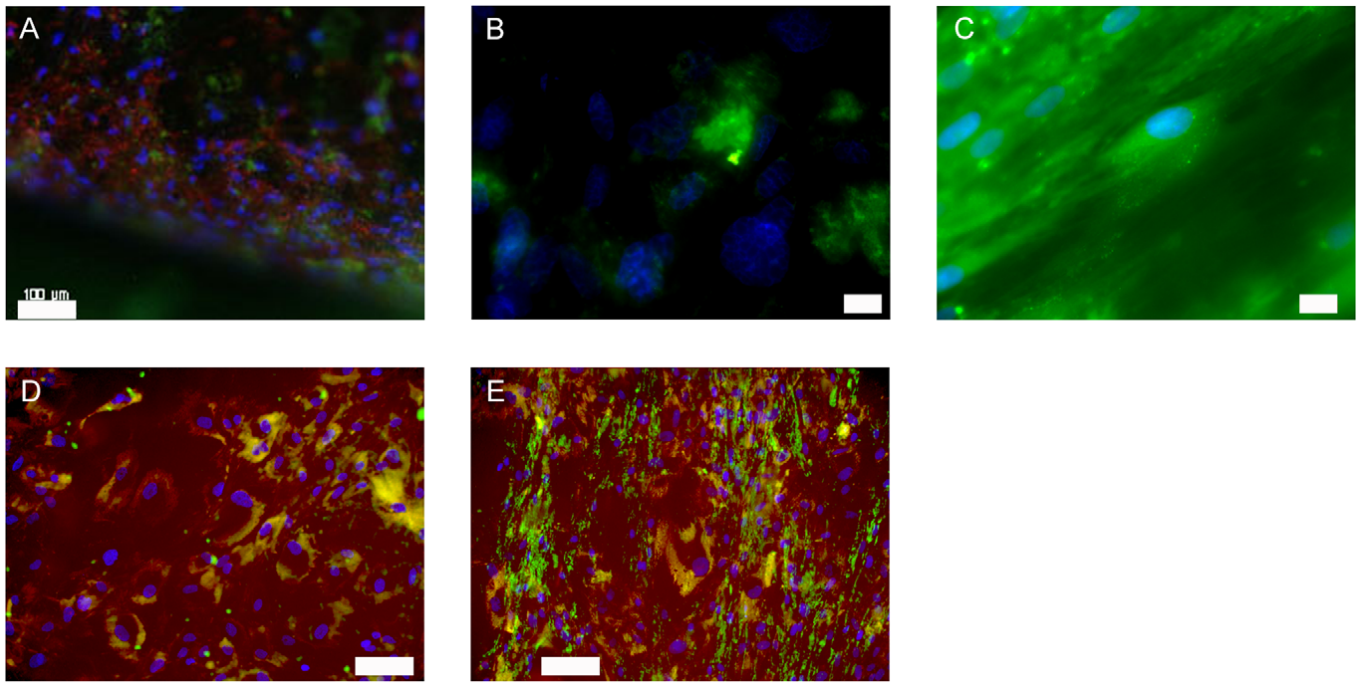
Expression of osteogenic markers in chemically differentiated ASCs on SLM-NiTi (A–C) and glass (D and E). A: green: BMP-6, red collagen type I, blue: nuclei; scale bar 100 µm. B: green: BMP-2, blue: nuclei; scale bar 10 µm. C: green: osteocalcin, blue: nuclei; scale bar 20 µm. D: green: collagen type I, red: osteocalcin, blue: nuclei: scale bar 80 µm. E: green: fibronectin, red: bone alkaline phosphatase, blue: nuclei; scale bar 100 µm.

To evaluate osteogenic marker expression, meshes were completely immunostained as indicated in table 1. Negative and positive controls on cover glasses were performed according to the same protocol. All investigated markers were positive in every sample type. A selection is shown in Figure 8. In the first row, BMP-6 expression is indicated by green fluorescence in osteogenically differentiated ASCs grown on meshes, while collagen I was positively stained red (Fig. 8A). Panel B visualized BMP-2 and panel C expression of osteocalcin both in green fluorescence. In the second row, osteogenic ASCs on cover glasses showed expression of osteocalcin (Figure 8D, red fluorescence) and collagen type I (green fluorescence). Figure 8E visualizes bone alkaline phosphatase in red fluorescence and fibronectin in green.

Undifferentiated cells served as negative controls. They were found to be positive for the mesenchymal marker proteins collagen type I and fibronectin, but not for bone markers bone alcaline phosphatase, osteoclacin, BMP-2, BMP-6, osteopontin and sparc (data not shown).

**Figure 9:**
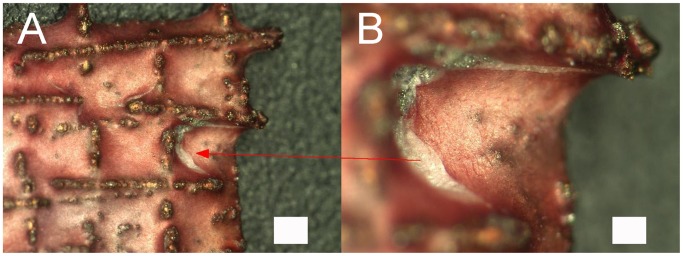
Alizarin red staining of chemically differentiated ASCs, 3d-digital microscopy. A: overview; scale bar 100 µm. B: detail; scale bar 20 µm.

Findings of immunofluorescence were confirmed by alizarin red stainings for calcium depositions (Fig. 9A and B): Osteogenically differentiated ASCs either grown on meshes or on cover glasses were found to be positive, whereas undifferentiated cells showed no staining.

**Figure 10:**
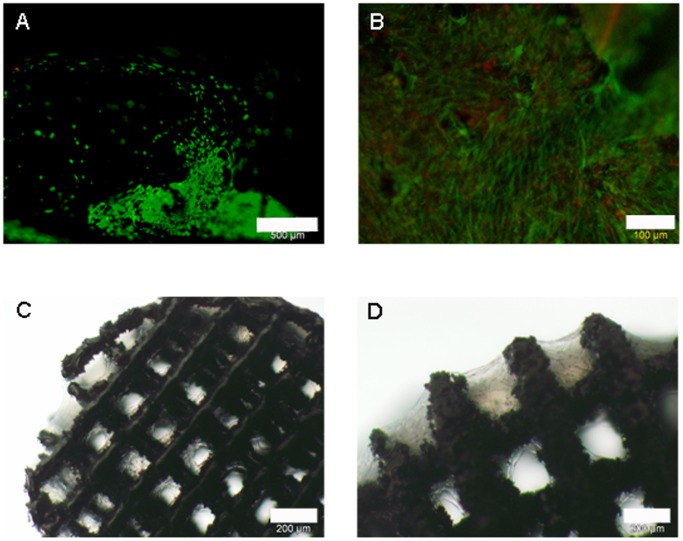
Mechanically differentiated osteogenic ASCs. A and B: live/dead staining; green: vital cells, red: dead cells; scale bars 500 µm (A), 100 µm (B). C and D: light microscopy; scale bars 200 µm (C), 300 µm (D).

As the previous results demonstrated ASCs could be differentiated osteogenically on SLM NiTi structures, we wanted to prove that three-dimensional cell growth in combination with mechanical force is able to induce osteogenesis without chemical manipulation. As demonstrated in figure 2, cages could be constructed with a defined inner structure. Cells were seeded on the cages for one week without applied forces to allow cell proliferation and migration into the inner parts of the cages. After that time, the cages were subjected to mechanical stress by compression of the SLM-structures. To evaluate the rates of living cells compared to controls we performed live/dead stainings. In spite of the high forces exerted continuously for six weeks, cell vitality was comparable to short time cultures on meshes and untreated controls (Fig. 10A and B, Fig. 4A). Analyzing the spaces inside the cage like structures it was clearly visible that ASCs grew inside the structures as well as on the outside (Fig. 10A and B, live/dead assay) and C and D (light microscopy). The lively green fluorescence which could be observed in the live/dead staining additionally indicates that cells did not only grow into the inner parts of the cages but remained vital (Figure 10A and B). This indicates that the implant design allows proper supply with O_2_ and nutrients.

**Figure 11:**
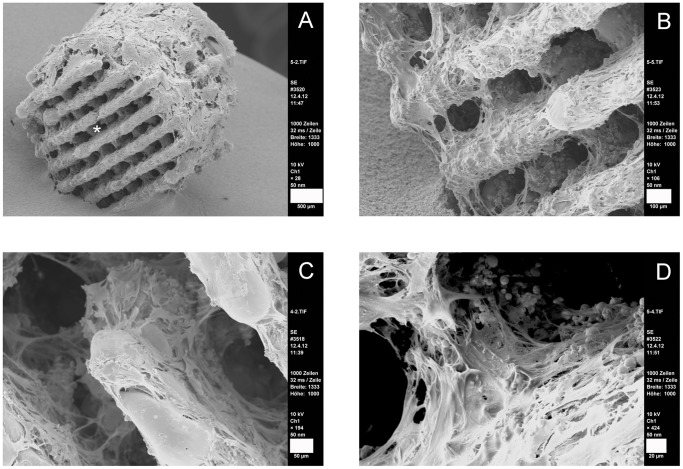
SEM of mechanically differentiated ASCs. A: overview; cells do not close the pores of the clamp-contact surface (indicated by asterisk); scale bar 500 µm. B–D: osteogenic ASCs inside the pores, scale bars 100 µm (B), 50 µm (C), 20 µm (D).

**Figure 12:**
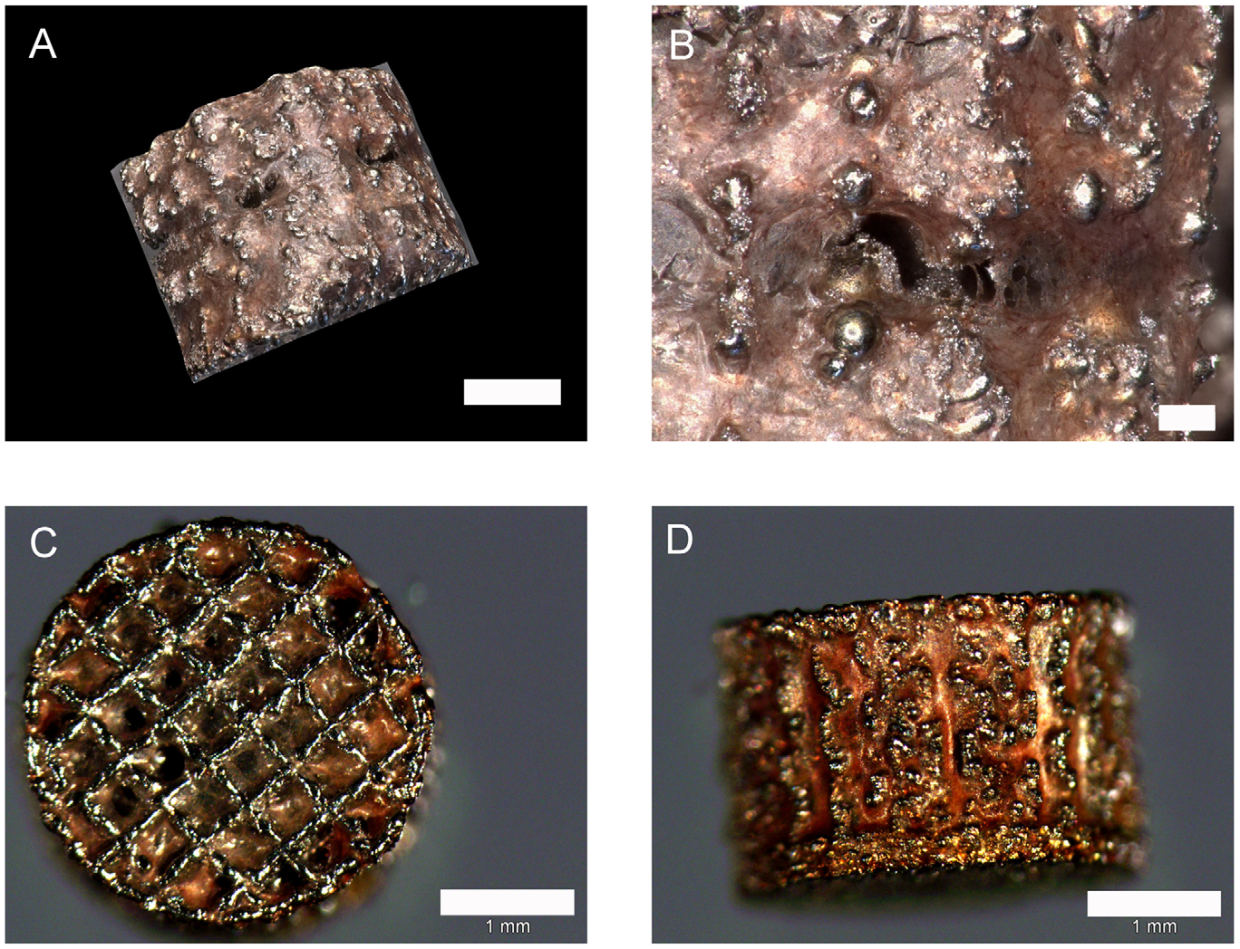
Alizarin red staining of mechanically differentiated ASCs. A and B: digital 3d-microscopy of rounded side; scale bars 1 mm (A), 150 µm (B) C and D: light microscopy of flat side (A) which was in contact to clamp and rounded side. (B); scale bars 1 mm.

The complete settling of cells was further confirmed by SEM pictures which gave a closer look into the cage pores (Fig. 11). Cells did not only grow inside the scaffolds but also developed a phenotype typical for osteogenic ASCs. This is visible by the flat and jagged morphology (Fig. 11C and D). Osteogenic differentiation was approved by alizarin red staining and immunofluorescence of osteogenic marker proteins. Evenly distributed calcium depositions were visualized by a homogenous red color (Fig. 12A–D) even deep inside the samples (note, the red signals inside the cages Figure legend). The staining was successful under all compression conditions even under the lowest.

**Figure 13:**
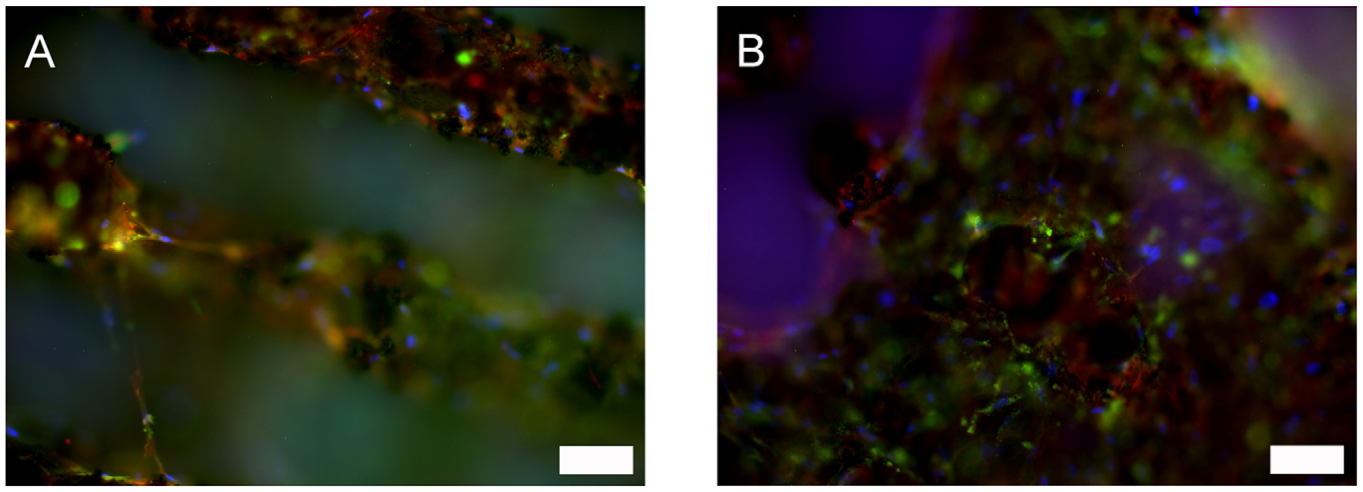
Expression of osteogenic markers in mechanically differentiated ASCs. A: green: osteocalcin, red: collagen type I, blue: nuclei; scale bar 100 µm. B: green: bone alkaline phosphatase, red: fibronectin, blue: nuclei; scale bar 100 µm.

Osteogenic markers could be positively detected as given in table 1. As examples osteocalcin (green fluorescence) and collagen I (red fluorescence) expression could be detected in cages subjected to a torque of 10 Nm over 6 weeks indicating that mechanical induction alone was sufficient to produce an osteogenic stimulus (Fig. 13A). Furthermore, we also verified expression of bone alkaline phosphatase (green fluorescence) and fibronectin (red fluorescence) (Fig. 13B).

When comparing the stressed specimen with untreated controls one has to realize that the cells do not close the pores of the contact surface by matrix secretion (Fig. 11A, asterisk) although the neighboring cells were vital. In contrast, the pores at the sides which were without contact to the titanium clamps were closed by cells and extracellular matrix (Fig. 11A). Further analyses are needed to evaluate to which extent the experimental set-up causes the described phenomenon.

Taken together these findings suggest the assumption that the mechanical induction of osteogenic differentiation leads to comparable results as the chemical induction.

### Calculation of Pressure Applied to SLM Structure

As there are many factors (friction, lubrication, surface roughness, material yield strength, surrounding conditions, etc.) influencing i.e. the friction coefficient [23], the calculation has to be understood as a rough estimation.
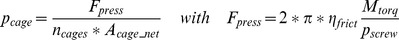



The tightening clamps were tightened by utilization of a torque wrench with a specific momentum (M_torq_). The resulting axial force (F_press_) can be calculated with the known screw pitch (p_screw_ of M8×1 = 1 mm) and friction coefficient (η_frict_ = 0.14 [24]).

To quantify the pressure applied to each SLM cage the resulting axial force has to be divided by the number of cages (n_cages_ = 3 per clamp) and the actual contact surface between clamp and SLM cage (A_cage_net_ = 3.74 mm^2^ per structure).

For osteogenic differentiation mechanical stress was applied by tightening the clamp screws with torque between 5 and 20 Nm. Approximated by the calculations above, these torque values correspond to 392 to 1568 N/mm^2^ pressure on each SLM cage.

## Discussion

### Viability and Growth Behavior

As creation of large pieces of bone still remains a challenge in vitro, use of hybrid-implants offers a bridge between tissue engineering and dense metal or ceramic implants.

Regarding flexibility and stability Nitinol is a promising material for bone implants. The SLM-processed alloy develops no negative influence on the cells. In comparison to glass or cell culture plastics no significant difference in viability was detected. This findings correlate with studies of Habijan, who analyzed effects of dense NiTi-surfaces on MSCs subjected to cyclic loading or in static culture [7]. He observed that MSCs were able to grow unaffectedly on NiTi-surfaces generated by injection molding or rolling. Surfaces generated by SLM seem to have positive properties for cell growth. ASCs shape on flat NiTi sheets was comparable to glass whereas SLM surfaces lead to a more dimensional cell shape as roughness of this kind of surfaces is higher. Hollander et al oberserved similar cell morphology of human primary osteoblast like cells from the iliac crest on TiAl6V4 discs processed by SLM. The vitality was comparable to flat TiAl6V4 discs, but cell density on SLM surfaces was clearly increased [25]. We also found high rates of life cells on SLM-NiTi surfaces comparable to flat glass or NiTi and an enhanced dimensional growth. Findings of Deliginanni regarding roughness of Ti alloy discs indicate a positive influence of rough surfaces on cell attachement founded in an enhanced protein binding to the surface [26].

The study at hand and studies of Dudziak et al. evaluated a good biocompatibily of SLM-NiTi –structures regarding blood cells and ASCs [20]. NiTi was in general found to have exellent biocompatibility characteristics [17], [19], [20], [27]. Recent studies raise questions. Jeswani et al. discussed the use of NiTi implants for intracranial stents. Based on clinical data they came to the conclusion that the use of NiTi stents for patients with known nickel allergies has to be considered carefully [28]. An in vitro study of Habijan showed that probably only a subpopulation of patients with nickel allergies reacts sensitive to NiTi [6]. Concerning to those data the SLM-NiTi implant needs to be evaluated further for allergic potential. Therefore testing under cyclic loading should be performed with a suitable experimental setup as it was developed by Habijan et al. [6], [29]. This will give informations about possible nickel release from the implant and allows studying cells reaction. Habijan tested commercially available dense NiTi cylindrical rods and found MSCs to survive on them although there was a slightly increased Nickel concentration in the cell culture media resulting from cyclic loading.

An in vivo model for testing the allergic potential of SLM-NiTi still remains difficult as there is only one rodent model available. Schmidt et al. generated mouses expressing the human variant of toll-like receptor-4 TRLR-4), which is responsible for allergic reactions to nickel [30]. The rodent variants of TLR-4 do not mediate immune responses to nickel.

### Osteogenic Differentiation without Chemical Stimulation

We could demonstrate for the first time that osteogenic differentiation of ASCs can be induced chemically as well as mechanically with similar results. So far mechanic differentiation of ASCs was performed within bioreactors combined with chemical stimulation, for example by supplementation with 10 nM dexamethasone 21-dihydrogen phosphate, which is a known osteogenic inducer [31]. These studies where performed on ceramics or glass [32], [33], [34]. The study at hand showed an independent way by application of forces via an implant without a chemical stimulation. ASCs were found to express several osteogenic markers when differentiation was induced mechanically. In comparison to chemical induced differentiation there were not found any visual differences in immunostainings or vitality analyses. Cells seeded on cage like structures grew inside and were still viable after 6 weeks. The inner structure of the cage-like implants seems to allow an appropriate supply with O_2_ and nutrients of the cells, which is confirmed by the high rates of live cells inside the structures. This is one of the striking points for long time survival of cells and in general for tissues lacking vascular supply [35]. The implant structures analyzed in this study seem to have optimal properties, according to Lee et al who showed that deep ingrowth and high rates of living cells in bone implant materials require interconnected pores with appropriate size [14]. Furthermore, they found lower viability and proliferation rates in scaffolds with random structured pores. Here the SLM technique has the crucial advantage to be able to process defined structures for cell growth directly from the computer model.

A continuative approach is the utilization of another NiTi-feature: the so called memory effect. In dental orthopedics the application of shape memory alloys is very popular, NiTi is used for example as orthodontic wire. As the study at hand demonstrated osteogenic differentiation of ASCs can be induced by application of mechanical stress. The memory effect of NiTi might be used to induce osteogenic. Inducing the memory effect by body-temperature during implantation leads to reshaping in term of a slight compression. This compression applies mechanical stress to the attached ASCs and is thought to lead to ostegenic differentiation.

Another trigger for osteogenic differentiation of ASCs after implantation is the bony environment. In a study of Lu et al. a bone biomimetic microenvironment could induce the osteogenic differentiation of ASCs. They tested a co-culture system of ASCs and human primary osteoblasts on 3d-nanocomposite scaffolds. ASCs were found to express high amounts of osteocalcin and also osteopontin after 14 days of co-cultivation [36].

### Further Prospects

Long time survival of our hybrid-implant in vivo depends on vascularization. The vascularization capacity of engrafted structures is one of major hurdles of tissue engineering [37]. Different strategies like use of endothelial cells or growth factors are pursued to obtain neo-vascularization of engineered tissues on scaffolds [38]. Unger et al. demonstrated that the combination of human osteoblasts and endothelial cells in vitro leads to a presettlement of porous NiTi with a microcapillary-like network [39]. Assad et al. achieved good results with a porous NiTi bone implant without cells in sheep. Within 12 months they found vessels grown into the pores of the implant [40]. Based on those data SLM NiTi cages offer a high potential to be supplied with vessels in vivo. As ASCs are a rich source of VEGF [41], [42] it is conceivable that there is no chemical loading of the implant needed to attract new vessels in a very short time. Based on the SEM analyses, cage-like implants should be implanted not later than 24 hrs after activation with autologous ASCs. A longer incubation results in an overgrowth of the pores by ASCs. Shorter in vivo culturing times or lower cell numbers are needed.

The system offers a fast way of therapy. Implant processing and cell withdrawal can be done within one day, ASCs settlement on the implant over night. So implantation can be executed the following day. This is a quite short time period for processing a complete customized implant. Implant integration might be supported by cells from surrounding bone tissue and marrow. The open structure allows the migration of cells from outside into the implant.

Due to the unavailability of NiTi-structures with an appropriate reset temperature by start of this study, the NiTi memory effect had to be simulated by external force application utilizing clamps (Fig. 1). The test system worked properly.

**Figure 14:**
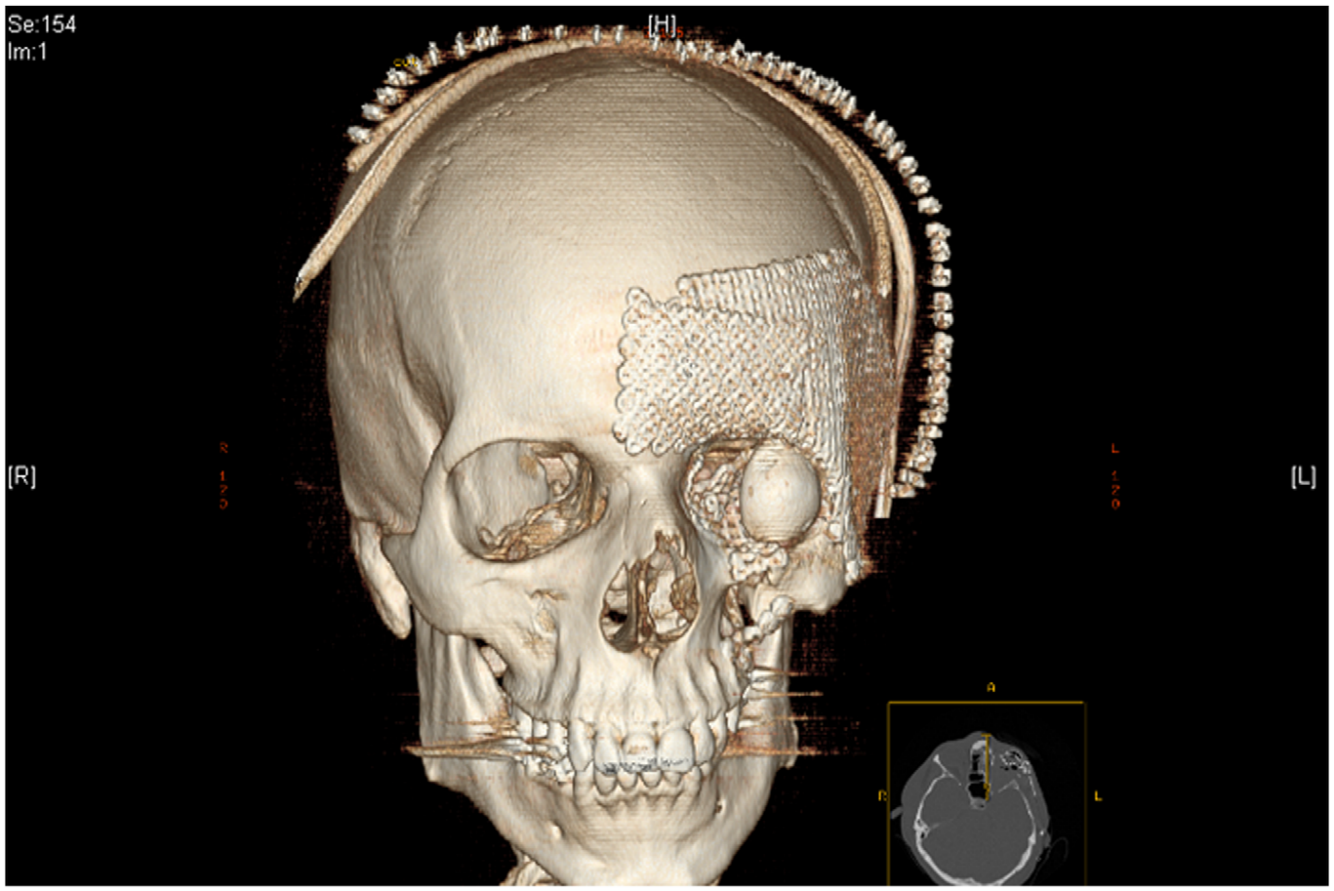
Possible application for NiTi-hybrid-implant (image created by Prof. Vogt and Prof. Gellrich, Hannover Medical School).

The low implant weight allows its application in every region of the body. It might be very useful for reconstructions in the face (Fig. 14) as well as for therapy of load bearing defects. The calculations of pressure revealed tolerances of more than 1568 N/mm^2^ of each cage. Turned to Kilogramms (m = F/g) this means a loadability of approximately 160 kg.

Although NiTi is extremely loadable, in long term disposition problems by material fatigue may occur. Several studies describe fractures of NiTi stents in vitro [43] and in vivo [44] as well as in dental instruments [45]. An elaborate discussion of fatigue and its causes in NiTi can be found in the review of Robertson et al. [46]. They postulate that fabrication, material processing and surface conditions have substantial influence on fatigue of NiTi, but in contrast to McKelvey et al. [47] Robertson et al. postulate that NiTi has no lower fracture toughness and faster crack growth than other implant materials. The discussion about fatigue of NiTi and the comparison to other approved implant materials is quite complex and controversial; nevertheless a fatigue of the implant especially in load bearing sides would be fatal. The developed SLM-generated implant has to undergo detailed investigations by experts to prevent failure after implantation.

### Forensic Snares

On account of the European Commission Directives 2006/17/EC [48] and 2006/86/EC [49] a license is needed to procure, process and if necessary store human autologous cells. Attending surgeons have to apply for a license by national institutions or make a draft on a tissue establishment. Nevertheless, our approach might be of clinical relevance as the use of certified kits and machines is covered by national legislations.

### Conclusion

SLM-fabricated 3d-micostructured NiTi-scaffolds combined with autologous ASCs offer new perspectives for customized osteoimplants especially for reconstruction of fine structures as necessary in the human face. As the problem of NiTi reset temperature has been solved [20], further studies have to reveal the necessary reset forces of the alloy for induction of osteogenic differentiation in detail. In animal experiments the possible ingrowth of blood vessels should be evaluated.
